# Longitudinal changes in processed food intake and their daily caloric contribution among Ghanaian populations living in Ghana and Europe: findings from the prospective Research on Obesity and Diabetes among African Migrants (RODAM) cohort study

**DOI:** 10.1186/s44263-025-00226-x

**Published:** 2026-01-02

**Authors:** Mustapha Titi Yussif, Reginald Adjetey Annan, Anthony Edusei, Mary Nicolaou, Erik Beune, Samuel Nkansah Darko, Ina Danquah, Karlijn A. C. Meeks, Ellis Owusu-Dabo, Charles Agyemang

**Affiliations:** 1 Department of Biochemistry and Biotechnology, Faculty of Biosciences, College of Science, Kwame Nkrumah University of Science and Technology, Kumasi, Ghana; 2 Department of Molecular Medicine, Kwame Nkrumah University of Science and Technology (KNUST), Kumasi, Ghana; 3 Department of Public and Occupational Health, Amsterdam Public Health Research Institute, Amsterdam University Medical Center, University of Amsterdam, Amsterdam, The Netherlands; 4 Division of Endocrinology, Diabetes and Nutrition, Department of Medicine, University of Maryland School of Medicine, Baltimore, MD USA; 5 Division of Endocrinology, Diabetes, and Metabolism, Department of Medicine, Johns Hopkins University School of Medicine, Baltimore, MD USA; 6 Heidelberg Institute of Global Health (HIGH), Universitätsklinikum Heidelberg, Medizinische Fakultät der Universität Heidelberg, Heidelberg, Germany; 7 Innovation for Planetary Health, Zentrum Für Entwicklungsforschung (ZEF), Universität in Bonn, Bonn, Germany; 8 School of Public Health, Kwame Nkrumah University of Science and Technology (KNUST), Kumasi, Ghana

**Keywords:** Processed foods, Ultra-processed foods, Energy, NOVA, Ghana

## Abstract

**Background:**

Dietary changes towards increased consumption of processed and ultra-processed foods are major contributors to obesity and non-communicable diseases worldwide. However, limited data exist on the intake of processed foods among Ghanaians living in Ghana and their migrant counterparts living in Europe. This study assessed changes in the intake of processed foods and their corresponding energy contributions among different Ghanaian populations.

**Methods:**

Data were collected from the Research on Obesity and Diabetes among African Migrants–prospective (RODAM-Pros) cohort study, which recruited Ghanaians living in rural and urban Ghana and Ghanaian migrants living in Amsterdam, the Netherlands between baseline (2011–2015) and follow-up (2019–2021). Dietary intake was assessed using standardized food frequency questionnaires. Foods were regrouped according to the NOVA classification, a system that categorizes food based on the degree of processing. Paired sample *t*-tests were used to determine the differences in mean daily intake (grams) of foods and energy contribution between baseline and follow-up.

**Results:**

Compared with baseline, ultra-processed food consumption decreased significantly among urban Ghanaians (−37.6%, −97.57 g/day, 95% confidence interval (CI) −129.66 to −65.48, *p* < 0.001) and migrants in Amsterdam (−31.1%, −168.90 g/day; 95%CI −241.54 to −96.26, *p* < 0.001) with no significant change in rural Ghana. The percentage of total energy from ultra-processed foods changed from 9.6 to 9.0% (*p* = 0.136) in rural Ghana, 15.9% to 13.9% (*p* < 0.001) in urban Ghana and 13.4% to 13.0% (*p* = 0.539) in Amsterdam. Conversely, processed food consumption increased across all sites: by 52.4% (+ 149.31 g/day, 95%CI127.70 to 170.92, *p* < 0.001) in rural Ghana, 31.4% (+ 96.67 g/day, 95%CI 75.15 to 118.19, *p* < 0.001) in urban Ghana and 29.0% (+ 62.91 g/day, 95%CI 44.18 to 81.63, *p* < 0.001) in Amsterdam. The percentage of total energy from processed food increased significantly from 20.8 to 39.5% (*p* < 0.001) in rural Ghana, 23.6% to 33.5% (*p* < 0.001) in urban Ghana and 7.2% to 12.9% (*p* < 0.001) among migrants.

**Conclusions:**

Across all Ghanaian population groups studied, dietary intake has shifted towards higher intake and caloric contribution of processed foods, while ultra-processed food consumption has declined in urban and migrant settings. Further research is needed to identify the drivers of these changes and their implications for cardio-metabolic health among Ghanaian populations.

**Supplementary Information:**

The online version contains supplementary material available at 10.1186/s44263-025-00226-x.

## Background

Processed and ultra-processed foods are mainly characterized by high energy density and low micronutrient content [1, 2]. As such dietary habits that favor processed food consumption pose a high threat to human health [3]. Evidence abounds that ultra-processed foods are linked to non-communicable diseases (NCDs) such as the obesity pandemic [4], cancer [5], cardiometabolic disorders [6, 7] among others, and mortality [8–11].

In sub-Saharan African (SSA) countries, there has been a rise in NCDs, which is believed to be driven in part by the nutrition transition where traditional diets are giving way to processed and ultra-processed food consumption which has been predicted to continue to rise in these countries if no policy interventions are implemented [12, 13].

Ghana, like many other SSA countries, has experienced dietary changes since the early 1990s [14], suggesting a progression into the later stages of the nutrition transition [15]. These changes have been linked to rising levels of obesity, hypertension, and cardiovascular diseases (CVDs), which vary across population subgroups by migration status and geographic location [16–18]. Understanding the role of processed foods in this transition is therefore critical.

In Ghana, the use of processed foods may be increasing under the influence of an obesogenic food environment [19, 20]. Even in rural areas, access to and consumption of processed and ultra-processed food may be rising due to a number of factors, including increased availability and changing lifestyles [21], greater incomes from non-farm jobs, mechanization of farm output, and an increase in demand for convenience [22]. There is also increasing urbanization, which could lead to increased consumption of processed foods as a result of the link between urbanization and higher incomes and employment opportunities. The growing time demands and the rise in the demand for convenience foods, which may be caused among other things by changes in the workforce and family patterns, notably the increasing economic participation of women, lead to a rise in demand for convenience foods [23].

In the advanced economies, findings from several observational studies reported a high intake of dietary energy from processed foods compared with unprocessed foods. For instance, in the United States, a previous study found that ultra-processed foods accounted for close to 58% of dietary energy among adults [24]. Similarly, ultra-processed foods contributed 55% of calories among Canadians [25]. Further, in the United Kingdom, a study revealed that 51% of calories were from ultra-processed foods [26]. Moreover, a large study in 10 European countries revealed that processed foods contributed 61–79% of energy intake in Spain, the Netherlands and Germany [27]. This suggests that Ghanaian migrants in Europe and other western countries may increase their consumption of processed foods due to dietary acculturation where they adopt diets that are higher in processed foods. However, little is known about the quantity of processed foods intake and the corresponding dietary energy intake from these processed foods and how these are changing over time. If the levels of dietary intake of processed foods and their trends among rural, urban and migrant Ghanaian groups are known then we can assess how the availability, affordability and marketing of processed foods differ within the different Ghanaian contexts. It will also help to assess the direct impact of urbanization and migration on diet and health outcomes. It would be known whether migrants retain their traditional diets or adopt host-country food patterns, and how these choices affect their health. Understanding these dietary trends can guide health interventions, policies, and educational programs to promote healthier eating habits among these populations.

Therefore, the present study assessed the changes in the intake of ultra-processed foods, processed foods, and unprocessed/minimally processed foods, and their corresponding daily caloric contribution across three different Ghanaian population groups living in rural and urban Ghana, and their migrant counterparts living in Amsterdam, the Netherlands over a 6-year period (baseline 2011–2015; follow-up 2019–2021).

## Methods

### Data source and collection

In this study we used data from the Research on Obesity and Diabetes among African Migrants (RODAM) prospective cohort study (RODAM-Pros) of which the details related to the participant recruitment process and data collection techniques have been published elsewhere [28, 29]. In brief, the RODAM-Pros cohort study was conducted to assess the drivers of the high burden of CVD risk by assessing the key changes in environmental exposures and epigenetic modifications among sub-Saharan African migrants. It is a longitudinal cohort study, which collected follow-up data between 2019 and 2021 from participants who completed the RODAM baseline study between 2011 and 2015 and were eligible for follow-up.

The RODAM study baseline [28] was based on a well-defined homogenous SSA population (i.e., Ghanaian migrants of mostly Akan ancestral heritage) living in three European cities (Amsterdam, the Netherlands; Berlin, Germany and London, UK) and their compatriots living in the rural and urban Ashanti region of Ghana. The RODAM-Pros cohort was restricted to Amsterdam, the Netherlands, rural and urban Ghana because the recruitment strategies in these sites allowed the study participants to be followed over time.

Two cities in Ghana (Kumasi and Obuasi) served as the sites for the urban areas whilst 15 villages in the Ashanti region served as the site for the rural participants. A multistage sampling procedure was used to arrive at 15 enumeration areas (EAs) for the rural sites and 15 EAs for the urban sites. Participants of Ghanaian migrants were randomly recruited for the Amsterdam study site from the Amsterdam Municipal register [28] which was accessed through the Amsterdam Municipal authority by the Amsterdam University Medical Centre and the Public Health Service (GGD) Amsterdam.

### Dietary assessment and classification of food groups

Dietary assessment was conducted using the 134-item Ghana Food Propensity Questionnaire (Ghana-FPQ) which queried for usual intake of foods in the past 12 months. The Ghana-FPQ is a questionnaire developed based on the European Food Propensity Questionnaire (EFPQ) [30] by incorporating typical Ghanaian foods identified from the 2008 Ghana Sociodemographic and Health Survey [31] as well as from previous studies conducted among urban-dwelling Ghanaians and Ghanaians living in Amsterdam [32].

Usual daily intake of foods in grams per day was estimated by combining the frequency of intake with standard portion sizes, which were determined through the conduct of 24-h dietary recalls among a random sub-sample of 251 participants for the Ghanaian food items and the EFPQ was used for the European foods [33].

We mapped and re-classified 30 food groups (grouping based on culinary use and nutrient profile) into four groups according to the NOVA international classification system which is based on the extent and purpose of processing [34]. The four groups were NOVA Group 1: unprocessed/minimally processed foods, NOVA Group 2: processed culinary ingredients, NOVA Group 3: processed foods, and NOVA Group 4: ultra-processed foods. The full list and constitution of the food groups extracted from the Ghana-FPQ and their respective NOVA classification are attached as Additional file 1: Table S1. Three food groups: vegetable soups, stews and sauces; meaty mixed dishes; and vegetarian mixed dishes were however not classified within the NOVA framework due to their complex composition.

The usual daily intakes in grams per day of the various NOVA classification groups were obtained by summing the intakes per day of food items within the respective food groups that constituted the NOVA group. Subsequently, the usual daily intakes (g/day) from each of the four NOVA groups were then translated into energy and nutrient intakes using the West African Food Composition Table (2012) [35] and the German Nutrient Database (BLS 3.01) (2010).

### Socio-demographic variables

Socio-demographic variables include sex, relationship status (married or registered partnership, cohabitating or living together, unmarried or never married, divorced or separated, widow/widower), educational status (categorized into four; never been to school or elementary school, lower vocational schooling or lower secondary schooling, intermediate vocational schooling or intermediate/higher secondary schooling, and higher vocational schooling or university), employment status (re-coded into two categories as employed and unemployed).

### Data analysis

Baseline data were summarized as means with standard deviations for continuous variables while categorical data were summarized using frequencies and their relative percentages. The normality of the absolute food intake (grams/day) and energy (kcals/day) was assessed using the Kolmogorov–Smirnov test where non-significant *p*-values (> 0.050) indicated normal distribution. We compared the mean daily intake of foods (grams/day) between baseline and follow-up using paired sample *t*-tests. The energy contribution relative to the total energy of each of the four food groups (unprocessed, culinary ingredients, processed, and ultra-processed foods) was analyzed stratified by the location of residence of the three Ghanaian populations and according to sociodemographic characteristics. The percentage of energy consumed from each food processing group was computed as the ratio of the energy intake from that food group to the total daily energy intake, and expressed as a percentage [36]. Although one NOVA group (processed culinary ingredients) was not presented separately in the results, its energy contribution was included in the denominator to ensure accurate computations. In addition, the energy contribution of the three unclassified food groups (listed in Additional file 1: Table S1) was also incorporated into the total daily energy intake used for percentage calculations.

The differences in the percentage of calories consumed from the various food groups between baseline and follow-up were also computed using the paired samples *t*-test. All statistical analyses were conducted using the statistical software IBM SPSS Statistics for Windows version 24.0 and the level of significance adopted was α ≤ 0.050.

## Results

The study involved a baseline sample of 2183 participants of predominantly Akan ethnic origin (83.4%) comprising rural and urban Ghanaian dwellers who make up 29.7% and 28.2%, respectively. The remaining 42.1% of the study sample was made up of Ghanaian migrants living in Europe (Amsterdam) with a mean length of stay of 18.8 years. Out of the 2183 participants, 569 (rural = 4, urban = 7, and Amsterdam = 558) did not have baseline dietary data while 102 did not have follow-up dietary data (loss to follow-up/non-response) comprising rural Ghana:10, urban Ghana:11 and Amsterdam:88 giving a dietary data follow-up response rate of 95.3%.

Table 1 shows the socio-demographic characteristics of the baseline study sample. The majority of the participants in rural Ghana were female (64.3%), married (58.9%), had only elementary education or had never been to school (60.9%) and were employed (84%) in the agricultural, forestry or fisheries sector (76.1%). The urban Ghana study site also had more female study participants (70.3%), more married people (63.7%) and (44.5%) had lower vocational/secondary schooling. Additionally, 84.7% of the participants living in urban Ghana were employed and mostly engaged in elementary occupations (42.3%) and the sales and services sector (22.8%). For Ghanaians living in Amsterdam, females constituted the greater proportion (60.7%) of the study participants. Furthermore, 29.7% of the Amsterdam participants were divorced or separated and another 29.7% had never been married while 37.6% of them had education up to lower vocational/secondary school with 34.3% of them being employed and mostly (71.8%) engaged in elementary occupations.

**Table 1:** Socio-demographic characteristics of study participants at baseline

Socio-demographic variable	Rural Ghana (*n* = 649) mean (SD)	Urban Ghana (*n *= 615) mean (SD)	Amsterdam (*n* = 919) mean (SD)
Age	47.8(13.1)	45.5(10.8)	45.7(10.2)
Household size	6.1(3.0)	5.1(2.4)	3.4(1.5)
Wealth Index	3.0(0.2)	3.0(0.2)	**–**
Length of stay in Europe (years)	N.A	N.A	18.8 (7.8)
*Sex*	*n (%)*	*n* (%)	*n* (%)
Male	232 (35.7)	179 (29.1)	361(39.3)
Female	417 (64.3)	436 (70.9)	558 (60.7)
*Relationship status*	*n (%)*	*n* (%)	*n* (%)
Married/registered partnership	359 (58.9)	381 (63.7)	162 (19.7)
Cohabiting (living together)	98 (16.1)	43 (7.2)	164 (19.9)
Unmarried (never married)	17 (2.9)	52 (8.7)	245 (29.7)
Divorced or separated	73 (12.0)	73 (12.2)	245 (29.7)
Widow/widower	63 (10.3)	49 (8.2)	8 (1.0)
*Religion*	*n (%)*	*n* (%)	*n* (%)
Christian	354 (82.7)	376 (83.6)	580 (83.2)
Islamic	36 (8.4)	61 (13.6)	26 (3.7)
Other religion or faith	38 (8.9)	13 (2.9)	91 (13.1)
*Occupation*	*n (%)*	*n *(%)	*n* (%)
Managers	2 (0.3)	6 (1.1)	1 (0.3)
Professionals	21 (3.6)	20 (3.5)	9 (2.8)
Technicians and associate professionals	0 (0.0)	14(2.5)	5 (1.5)
Clerical support workers	1 (0.2)	14 (2.5)	17 (5.3)
Service and sales workers	35 (6.1)	130 (22.8)	42 (13.0)
Skilled agric, forestry, fishery	439 (76.1)	20 (3.5)	0 (0.0)
Craft and related trades workers	27 (4.7)	101 (17.7)	6 (1.9)
Plant and machine operators	2 (0.3)	24 (4.2)	11(3.4)
Elementary occupations	50 (8.7)	241 (42.3)	232 (71.8)
*Education*	*n (%)*	*n* (%)	*n* (%)
Never been to school or elementary schooling only	371 (60.9)	227 (37.8)	261 (31.0)
Lower vocational schooling or lower secondary schooling	188 (30.9)	267 (44.5)	317 (41.1)
Intermediate vocational schooling or intermediate/higher secondary schooling (general)	37 (6.1)	76 (12.7)	207 (37.6)
Higher vocational schooling or university	13 (2.1)	30 (5.0)	58 (6.9)
*Employment status*	*n (%)*	*n* (%)	*n* (%)
Employed	545 (84.0)	521 (84.7)	315 (34.3)
Unemployed	104 (16.0)	94 (15.3)	604 (65.7)

Data are reported as frequency (percentage) and mean (standard deviation); *SD *standard deviation, *NA *not applicable, – variable not collected

### Changes in daily consumption of unprocessed, processed, and ultra-processed foods

The consumption of unprocessed/minimally processed foods among Ghanaians living in rural Ghana showed a significant decrease (*p* < 0.001) by −27.8% (mean difference −334.08 g/day; 95% confidence interval (CI) −419.00 to −249.17) from baseline intake and follow-up (Table 2). Conversely, for urban-dwelling Ghanaians, the mean daily consumption of unprocessed/minimally processed foods increased significantly by 10.1% (mean difference:118.2 g/day; 95%CI 65.05 to 171.37, *p* < 0.001) while there was no significant change among Ghanaian migrants living in Amsterdam.

**Table 2:** Changes over time in mean total daily intake (grams) of different levels of processed foods according to study site

Site		Unprocessed/minimally processed food				Processed foods					Ultra-processed foods		
		Mean intake (g/day)	Mean Diff. (95%CI)	% Change	*p*-value	Mean intake (g/day)	Mean Diff.(95%CI)	% Change	*p*-value	Mean intake (g/day)	Mean Diff.(95%CI)	% Change	*p*-value
Rural Ghana	Baseline	1537.44	−334.08 (−419.00 to −249.17)	−27.8	< 0.001	284.95	149.31 (127.70 to 170.92)	52.40	< 0.001	164.49	−17.93 (−55.42 to 19.57)	−10.90	0.348
	Follow-up	1203.36				434.26				146.56			
Urban Ghana	Baseline	1051.64	118.21 (65.05 to 171.37)	10.10	< 0.001	307.59	96.67 (75.15 to 118.19)	31.43	< 0.001	259.70	−97.57 (−129.66 to −65.48)	−37.57	< 0.001
	Follow-up	1169.85				404.26				162.13			
Amsterdam	Baseline	2140.24	−49.28 (−185.04 to 86.47)	−2.30	0.476	217.18	62.91 (44.18 to 81.63)	28.97	< 0.001	542.52	−168.90 (−241.54 to −96.26)	−31.13	< 0.001
	Follow-up	2090.96				280.09				373.62			

*Mean Diff. (95%CI)* mean difference (95% confidence interval) and paired sample *t* test *p*-value. Mean difference calculated as mean daily nutrient intake (grams) at follow up–mean daily nutrient intake (grams) at baseline

For processed foods, participants from rural Ghana increased their consumption by 52.40% (mean difference:149.31 g/day; 95% CI 127.70 to 170.92, *p* < 0.001). Similarly, increased consumption of processed foods was observed among participants from urban Ghana (31.43% increase, mean difference 96.67 g/day; 95%CI 75.15 to 118.19, *p* < 0.001) and migrants living in Amsterdam (28.97% increase, mean difference 62.91 g/day; 95%CI 44.18 to 81.63, *p* < 0.001).

The daily intake of ultra-processed foods on the other hand remained unchanged over the period for participants in rural Ghana. Whereas reduced intake of ultra-processed foods was observed among participants from urban Ghana (37.57% decrease, mean difference −97.57 g/day; 95%CI −129.66 to −65.48, *p* < 0.001) as well as among migrant participants living in Amsterdam (31.13% decrease, mean difference −168.90 g/day, 95%CI −241.54 to −96.26, *p* < 0.001).

### Changes in caloric contribution of unprocessed, processed and ultra-processed foods

After 6 years (± 0.6) of follow-up, the estimated percentage of total daily energy from the consumption of unprocessed/minimally processed foods by participants in rural Ghana significantly decreased from 56.8 to 44.9% (% change −11.9%, 95%CI −14.7 to −9.1, *p* < 0.001) whereas the percentage of total daily energy from processed foods significantly increased from 20.8 to 39.5% (% change 18.8%, 95%CI 17.4 to 20.1, *p* < 0.001). However, the percentage of total energy from the consumption of ultra-processed foods did not significantly change (% change −0.6%, 95%CI −1.4 to 0.2, *p* = 0.136) among the rural dwellers in Ghana.

Similarly, the estimated percentage of total daily energy from the intake of unprocessed/minimally processed foods among the Urban Ghana cohort significantly decreased from 53.9 to 41.9% (% change −12.0%, 95% CI −14.2 to −9.7, *p* < 0.001) while that of processed foods increased significantly from 23.6 to 33.5% (% change: 9.9%, 95%CI 8.7 to 11.2, *p* < 0.001). There was a significant decrease in the percentage of total daily energy from ultra-processed foods (% change: −2.0%, 95%CI −3.0 to −1.1, *p* < 0.001).

For Ghanaian migrants in Amsterdam, the proportions of total daily energy from unprocessed/minimally processed foods and ultra-processed foods did not vary significantly between baseline and follow-up. On the other hand, a significant increase from 7.2 to 12.4% (% change from baseline intake: 5.3%, 95%CI 44.3 to 66.3, *p* < 0.001) was observed in the percentage of total daily energy from the consumption of processed foods among the Amsterdam group (Table 3).

**Table 3:** Changes in percentage of total daily energy intake from the different levels of processed foods among different Ghanaian populations

		Unprocessed/minimally processed foods			Processed foods			Ultra-processed foods		
		% of total daily energy	% Change (95%CI)	*p*-value	% of total daily energy	% Change (95%CI)	*p*-value	% of total daily energy	% Change (95%CI)	*p*-value
Rural Ghana	Baseline	56.8	−11.9 (−14.7 to −9.1)	< 0.001	20.8	18.8 (17.4 to 20.1)	< 0.001	9.6	−0.6 (−1.4 to 0.2)	0.136
	Follow up	44.9			39.5			9.0		
Urban Ghana	Baseline	53.9	−12.0 (−14.2 to −9.7)	< 0.001	23.6	9.9 (8.7 to11.2)	< 0.001	15.9	−2.0 (−3.0 to −1.1)	< 0.001
	Follow up	41.9			33.5			13.9		
Amsterdam	Baseline	27.4	−0.2 (−2.0 to 1.6)	0.828	7.2	5.3 (4.3 to 6.3)	< 0.001	13.4	−0.4 (−1.9 to1.0)	0.539
	Follow up	27.2			12.4			13.0		

Data are presented as mean percentage of total daily energy.* Mean Diff. (95%CI) *mean percentage change (95% confidence interval); paired samples *t *test *p*-value. Mean percentage change is calculated as Mean percentage of total energy (%) at follow up–mean percentage of total energy (%)) at baseline

An analysis of the socio-demographic drivers of the changes in the daily caloric contribution of the various levels of processed food shows that, for unprocessed/minimally processed foods, the decrease in caloric contribution occurred across sexes, educational level, relationship and employment status with the exception of unmarried people (*p *= 0.287) and tertiary level participants (*p* = 0.289) in rural Ghana. In urban Ghana, the decreased caloric contribution of unprocessed/minimally processed foods occurred across all socio-demographic groups while in Amsterdam, no significant change in caloric contribution was recorded across all groups with the exception of participants with secondary school/intermediate level of education whose caloric contribution from unprocessed foods increased by 3.2% (Additional file 1: Table S2). The caloric contribution of processed foods increased across all study sites and irrespective of socio-demographic status with the exception of Amsterdam where the widowed (*p* = 0.287) and university level participants (*p* = 0.372) had unchanged daily caloric contribution of processed foods between baseline and follow-up (Additional file 1: Table S3).

For ultra-processed foods, their caloric contribution was unchanged in Amsterdam and rural Ghana across all socio-demographic groups whereas in urban Ghana, the decrease in caloric contribution was observed only among males (−3.4%), married people (−2.9%) and people with secondary school educational level (−3.7%) (Additional file 1: Table S4).

### Change in daily caloric contribution of individual food groups

The daily caloric contribution of most of the individual food groups changed significantly between baseline and follow-up for each of the study sites with the exception of sweet spreads, alcoholic beverages and peanuts (Table 4).

**Table 4:** Change in daily caloric contribution of individual food groups

Food groups	Rural Ghana		Urban Ghana		Amsterdam	
	Mean % change (95% CI)	*p*-value	Mean % change (95% CI)	*p*-value	Mean % change (95% CI)	*p*-value
Whole grains and cereals	1.07 (0.81 to 1.33)	**< 0.001**	1.84 (1.42 to 2.25)	**< 0.001**	−0.89 (−1.23 to −0.54)	**< 0.001**
Refined cereals	−3.57 (−4.41 to −2.72)	**< 0.001**	−5.10 (−5.83 to −4.36)	**< 0.001**	**0**.85 (0.38 to 1.31)	**< 0.001**
Sweet spreads	−0.002 (−0.01 to 0.006)	0.618	0.01 (−0.01 to 0.03)	0.324	0.03 (−0.15 to 0.07)	0.196
Dairy products	−0.55 (−1.13 to 0.03)	0.065	0.84 (0.03 to 1.66)	**0.043**	0.89 (−0.32 to 2.09)	0.151
Fruits	−4.23 (−4.96 to −3.50)	**< 0.001**	0.52 (0.10 to 0.95)	**0.015**	2.67 (2.03 to 3.30)	**< 0.001**
Nuts and seeds	−0.14 (−0.36 to −0.08)	0.213	−0.23 (−0.45 to −0.01)	**0.037**	1.20 (0.93 to 1.47)	**< 0.001**
Roots, tubers and plantains	−4.03 (−5.33 to −2.73)	**< 0.001**	0.58 (−0.21 to 1.37)	0.153	0.53 (0.12 to 0.95)	**0.012**
Potatoes	−0.17 (−0.36 to 0.02)	0.080	0.17 (0.09 to 0.24)	**< 0.001**	0.10 (−0.04 to 0.23)	0.151
Fermented maize products	23.98 (22.97 to 25.00)	**< 0.001**	18.21 (17.21 to 19.22)	**< 0.001**	3.97 (3.32 to 4.62)	**< 0.001**
Vegetables	−0.47 (−0.68 to −0.27)	**< 0.001**	0.24 (−0.02 to 0.500)	0.072	0.59 (0.35 to 0.82)	**< 0.001**
Legumes	−1.00 (−1.35 to −0.66)	**< 0.001**	−0.71 (−1.07 to −0.35)	**< 0.001**	−0.04 (−0.22 to 0.15)	0.702
Rice and pasta	−1.55 (−2.05 to −1.05)	**< 0.001**	−2.86 (−3.43 to −2.29)	**< 0.001**	0.41 (0.03 to 0.78)	**0.033**
Egg	0.28 (0.20 to 0.36)	**< 0.001**	0.42 (0.31 to 0.52)	**< 0.001**	0.22 (0.15 to 0.30)	**< 0.001**
Red meat	−0.60 (−0.87 — −0.33)	**< 0.001**	−1.49 (−1.81 to −1.17)	**< 0.001**	0.05 (−0.17 to 0.28)	0.645
Poultry	−0.30 (−0.43 to −0.18)	**< 0.001**	−0.19 (−0.31 to −0.07)	**< 0.001**	0.42 (0.22 to 0.62)	**< 0.001**
Processed meat	−0.10 (−0.21 to 0.002)	0.054	−0.35 (−0.51 to −0.20)	**< 0.001**	0.05 (−0.08 to 0.19)	0.419
Fish	−1.37 (−1.57 to −1.18)	**< 0.001**	−1.60 (−1.86 to −1.35)	**< 0.001**	0.06 (−0.10 to 0.23)	0.443
Cakes and sweets	0.46 (0.18 to 0.73)	**0.001**	−0.98 (−1.23 to −0.73)	**< 0.001**	−0.10 (−0.32 to 0.12)	0.379
Coffee and tea	−6.78 (−8.19 to −5.36)	**< 0.001**	−7.30 (−8.83 to −5.76)	**< 0.001**	−9.84 (−12.59 to −7.09)	**< 0.001**
Alcoholic beverages	−0.08 (−0.22 to −0.07)	0.296	−0.05 (−0.11 to 0.01)	0.109	−0.05 (−0.28 to 0.19)	0.697
Sodas and Juices	−0.16 (−0.54 to 0.22)	0.416	−1.59 (−2.00 to −1.18)	**< 0.001**	−1.01 (−1.40 to −0.63)	**< 0.001**
Palm oil	−0.61 (−0.73 to −0.50)	**< 0.001**	−0.19 (−0.28 to −0.09)	**< 0.001**	0.04 (0.01 to 0.07)	**0.014**
Olive oil	−0.004 (−0.004 to 0.0119)	0.339	0.02 (0.004 to 0.395)	**0.016**	0.14 (0.08 to 0.20)	**< 0.001**
Peanut	0.08 (−0.06 to 0.21)	0.272	−0.03 (−0.21 to 0.16)	0.756	0.17 (−0.18 to 0.53)	0.332
Margarine	−0.26 (−0.41 to −0.12)	**< 0.001**	**−**0.28 (−0.42 to −0.14)	**< 0.001**	−0.20 (−0.32 to −0.09)	**0.001**
Cooking fats	−0.0025 (−0.0040 to −0.0011)	**0.001**	0.0006 (−0.0005 to 0.00170)	0.285	−0.00014 (−0.00087 to 0.00059)	0.705
Condiments	−0.27 (−0.43 to −0.12)	**0.001**	−0.31 (−0.47 to −0.16)	**< 0.001**	−0.76 (−1.15 to −0.38)	**< 0.001**

Bold values are significant at *p* < 0.05

For the rural Ghana study site, the daily caloric contribution of the individual food groups with the highest changes was fermented maize products and whole grains and cereals, which increased by 24.0% and 1.1% respectively. Also, coffee and tea and fruits recorded the greatest significant reduction in caloric contribution to daily energy among this group by 6.8% and 4.2% respectively. Similarly, fermented maize products and whole grains and cereals recorded the highest significant increases in caloric contribution to daily energy consumption of 18.2% and 1.2% respectively among urban dwellers while the highest significantly decreased caloric contributions were recorded by coffee and tea (7.3%) and refined cereals (5.1%).

Ghanaian migrants living in Amsterdam recorded 4.0% and 2.7% respectively for fermented maize products and fruits, representing the highest significant increases in daily caloric contribution whereas coffee and teas (9.8%) as well as sodas and juices (1.0%) represented the individual food groups with the highest significant decrease in daily caloric contribution.

## Discussion

The findings from this study show a decline in the proportion of energy consumed from unprocessed/minimally processed food in both rural and urban Ghana, and no changes among migrant Ghanaians living in Amsterdam. A simultaneous increase in processed food consumption and in the contribution of processed foods to total energy intake was observed in each population group, confirming a common trend toward greater dependence on processed foods. The percentage of energy from ultra-processed foods among rural and migrant Ghanaians did not change, whereas the energy contribution from ultra-processed food declined for urban Ghanaians.

The findings revealed that the quantities of intake of unprocessed/minimally processed foods did not significantly change among Ghanaians living in Amsterdam resulting in an unchanged caloric contribution of these foods to total daily energy. On the other hand, consumption significantly decreased among individuals living in rural Ghana resulting in a decrease in the percentage of energy from these foods. In contrast, there was a significant increase in the quantity of unprocessed foods consumed among Ghanaian urban dwellers which did not lead to an increase in the percentage of energy but rather a decline. This decrease in the percentage of energy from unprocessed/minimally processed foods among rural and urban Ghanaians was observed with a corresponding increase in the percentage of energy from processed foods which was mainly contributed by traditionally processed food items such as cereals. This phenomenon reflects evolving dietary practices among these groups; however, the shift does not align with the typical pattern of the nutrition transition which is mostly characterized by dependence on industrially processed and ultra-processed foods [37]. The decreased percentage of energy from unprocessed/minimally processed food among the urban dwellers in spite of their increased consumption could be a result of the increase in the overall caloric intake. It also seems possible that the unprocessed/minimally processed foods consumed by this group may be in a lower-energy form thus resulting in the decreased percentage of energy from these foods.

A study by Cattafesta et al. [38] in Brazil among rural farmers ranked ultra-processed foods as one of the largest contributors to total calories (17.7%), being second to unprocessed/minimally processed foods (64.7%). While this study does not explicitly indicate a reduction in the consumption of unprocessed/minimally processed foods over time, it demonstrates that rural diets, which hitherto were largely made up of unprocessed/minimally processed foods, now feature a significant proportion of processed foods. This position is supported by another recent piece of evidence from a synthesis review of food systems transformations conducted by Baker et al. [39] which points to the issue of home-grown foods and unprocessed foods giving way to processed foods in rural areas in Africa and Asia due to increasing exposure to supermarkets and packaged food.

Our study also found that the daily consumption levels of processed foods have increased across all the different Ghanaian populations translating into an increased energy percentage contribution of processed foods to total energy of 19% among rural dwellers, 10% among urban dwellers and 5% among migrant Ghanaians in Amsterdam. Our findings of the increased daily consumption of processed foods among the different Ghanaian populations are largely supported by previous findings that there is a rise in the consumption of processed foods across West African countries [40].

The notable differences in the change of consumption of unprocessed and processed foods across geographical locations likely reflect the large role of contextual environmental factors in shaping what people eat. Previous studies have shown that there is robust marketing and increased access to processed and foods [41–43] in especially urban areas leading to an over consumption of energy from these foods at the expense of unprocessed/minimally processed freshly prepared meals [44]. Also, it has been noted in Ghana that, processed foods account for the largest share of all foods in the urban food environment where 80% of retail outlets sell processed foods [45]. Processed foods also constitute the most dominant food category in retail outlets making it highly accessible in equal measure as ultra-processed foods [45]. For the increase in the intake of processed foods and the rise in the percentage of calories from these foods in rural areas in Ghana, this may be as a result of factors such as increased incomes from non-farm activities as well as increased mechanization of farm production and the situation where more women are now working outside their homes as suggested by the International Food Policy Research Institute (IFPRI) [46]. Further, in rural areas in Ghana, processed staple foods such as processed maize meals, gari (grated cassava), smoked fish among others may also be cheaper than imported packaged snacks and frozen meals [47] making people rely more on the traditionally processed foods instead. Previous studies have also shown that migrants in Europe of Ghanaian origin mostly retain their traditional food choices [48], which mostly involve the use of processed ingredients such as dried fish, gari, etc.; however, these ingredients may be replaced with more processed options such as tomato paste and canned fish among others due to availability.

With respect to ultra-processed foods, whereas consumption levels remained unchanged for the rural Ghana population and their migrant counterparts, there was a decline in consumption and percentage contribution to total energy among the urban Ghanaian population. In contrast to these findings where the percentage of energy from ultra-processed foods among urban dwellers in Ghana declined from 15.9 to 13.9%, an almost concurrent study conducted between 20082009 and 2009 and 2017 and 2018 in Brazil using data from national household budget surveys showed an increase in the percentage energy from ultra-processed foods in urban areas from 19.94 to 20 0.55% [49]. The differences in dietary recall periods, the type of data used, and characteristics of the study subjects may have partly accounted for the different patterns of caloric intake from ultra-processed foods in the two studies. Most of the previous studies that have observed increasing consumption levels of ultra-processed foods in urban areas have cited various reasons including aggressive marketing [41], increased physical availability [42], and affordability [43].

There is a global rise in the consumption of ultra-processed foods as they already constitute the highest contributors to daily calories in high-income countries such as the United Kingdom [50], the USA [51], Canada [52], and Australia [53]. On the contrary, we noted that the proportion of total energy contributed by ultra-processed foods among the Ghanaian population living in Amsterdam, the Netherlands, a high-income country has not significantly increased over the period. We hypothesize that the apparent stagnation in the percentage of calories from ultra-processed foods among this group of migrants may be related to a saturation effect where the consumption of ultra-processed foods has stabilized after an initial high increase when they first became exposed to the Dutch food environment. Otherwise, this could be due to a slower change in food consumption patterns inspired by a lower level of acculturation [48].

This study provides valuable insights into the longitudinal changes in processed food intake among populations experiencing rapid dietary changes. The strengths include its prospective design, and stratification of the study population according to rural, urban and migrants, which helped to assess the environmental influence on food and energy intake. Another strength is that there was standardization of the data collection process across study sites where the same methods were used in Amsterdam, rural and urban Ghana. We also used individual-level dietary survey data, which estimated actual food consumption unlike other studies that used food availability and statistics of production and trade, which may not provide a completely accurate reflection of dietary consumption. The use of the NOVA food classification system is also a plus since it has been recognized by UN agencies as a relevant approach for linking dietary intake, obesity and NCDs [44, 54, 55].

This study, however, is not without limitations. First, as may be the case for most dietary studies, the limitation of self-reporting and recall bias leading to misreporting cannot be ruled out in this study. Its effect however on the study outcomes may be minimal due to the standardization of the data collection process across the sites. Secondly, the original dietary survey was not designed specifically to categorize foods according to characteristics of industrial processing, and so some misclassification of foods at the individual level cannot be excluded given that we applied the classification at the level of food groups rather than individual food items, which could lead to some overestimation or underestimation of the energy contribution from the various food groups. However, we minimized classification errors by setting standardized, objective and clear criteria, and a conservative approach (assigning a lower level of processing) was used in case of uncertainty.

## Conclusions

This study concludes that dietary changes are occurring within population groups in Ghana with a decreased intake of unprocessed foods and increased consumption of processed foods largely from fermented corn products. While this trend reflects some elements of the nutrition transition, the increased processed food consumption is based on traditional preparations and may not be entirely harmful. Nonetheless, this dietary shift signals a departure from fresher, whole foods and may open pathways to increased intake of ultra-processed foods that may compromise long-term health. Our findings indicate that the rate of increase in processed food consumption differs by geographical location, suggesting an important influence of contextual factors. This underscores the need for targeted strategies such as educational campaigns and subsidies for fresh foods among others to curb the rising intake of these potentially harmful foods. Further research is necessary to identify more specific contextual drivers of this shift in dietary intake to inform the development of tailored interventions.

## Supplementary Information

Supplementary material 1: Table S1: Description of food groups and NOVA classification. Table S2: Changes in percentage of total daily energy intake of unprocessed/minimally processed foods according to study site and socio-demographic factors. Table S3: Changes in percentage of total daily energy intake of processed foods according to study site and socio-demographic factors. Table S4: Changes in percentage of total daily energy intake of ultra-processed foods according to study site and socio-demographic factors.

## Data Availability

The RODAM-Pros data has been deposited on Zenodo [10.5281/zenodo.17520713] [56]. However, in order to preserve participant anonymity and comply with the ethical approvals and confidentiality agreements for this study, the data are not publicly available and can be accessed upon request from the investigators. Requests to access the dataset used in these analyses should be directed to the RODAM Study Coordinator (rodamcoordinator@amsterdamumc.nl). The analytical procedures for this particular study were performed using SPSS, and no programming codes were generated.
