# Supplementary material for: Longitudinal changes in processed food intake and their daily caloric contribution among Ghanaian populations living in Ghana and Europe: findings from the prospective Research on Obesity and Diabetes among African Migrants (RODAM) cohort study
Source: BMC Glob Public Health. 2026 Jan 2;4:2. doi: 10.1186/s44263-025-00226-x (PMC12763962; doi:10.1186/s44263-025-00226-x)
Supplement: Supplementary file 1 — Supplementary material 1: Table S1: Description of food groups and NOVA classification. Table S2: Changes in percentage of total daily energy intake of unprocessed/minimally processed foods according to study site and socio-demographic factors. Table S3: Changes in percentage of total daily energy intake of processed foods according to study site and socio-demographic factors. Table S4: Changes in percentage of total daily energy intake of ultra-processed foods according to study site and socio-demographic factors. [file 44263_2025_226_MOESM1_ESM.docx]

Table S1: Description of food groups and NOVA classification

| **Food group** | **Description** | **Processing Levels according to NOVA Classification** |
| --- | --- | --- |
| Whole grains and cereals | Whole grain bread, wholegrain crispbread, muesli cereals, and other grains (millet, couscous, polenta, spelt, and barley) | G1: Unprocessed/minimally processed |
| Fruits | Orange, mandarin, kiwi, watermelon, mango, cantaloupe, pawpaw, pineapple, banana, plum, peach, apricot, nectarine, flat peach, apple, pear, strawberries, cherries, berries, grapes, and stewed fruit | G1: Unprocessed/minimally processed |
| Peanut | Peanut, peanut butter | G1: Unprocessed/minimally processed |
| Nuts and seeds | Dried fruit, nuts, and seeds | G1: Unprocessed/minimally processed |
| Roots, tubers and plantain | Plantain, cassava, yam, and fufu | G1: Unprocessed/minimally processed |
| Potatoes | Potatoes, pan fried potatoes, French fries, and sweet potatoes | G1: Unprocessed/minimally processed |
| vegetables | Green leaves, spinach, chard, lettuce, endive, chicory, Chinese and white cabbage, tomatoes, peppers, carrots, cucumber, eggplant, beans (green beans), onions and garlic | G1: Unprocessed/minimally processed |
| Legumes | Groundnut soup, legumes, lentil-pea and bean soup | G1: Unprocessed/minimally processed |
| Egg | Egg | G1: Unprocessed/minimally processed |
| Red meat | Beef, goat, pork, game, liver, and giblets | G1: Unprocessed/minimally processed |
| Poultry | Poultry | G1: Unprocessed/minimally processed |
| Fish | Fatty fish, lean fish, fish preparations and shellfish | G1: Unprocessed/minimally processed |
| Coffee and tea | Regular coffee, decaffeinated coffee, black and green tea, and fruit and herbal tea | G1: Unprocessed/minimally processed |
| Olive oil | Olive oil | G2: Processed culinary ingredients |
| Palm oil | Palm oil | G2: Processed culinary ingredients |
| Cooking fats | Cooking fats (e.g. animal fats like lard or speck) | G2: Processed culinary ingredients |
| condiments | Ketchup, mayonnaise, crème fraiche, salad cream, sour cream, remoulade, and sauces | G2: Processed culinary ingredients |
| Fermented maize products | Banku and kenkey | G3: Processed foods |
| Refined cereals | White wheat bread, white crispbread, hot cereals, and porridge | G3: Processed foods |
| Rice and pasta | Rice, pasta, noodles, and macaroni | G3: Processed foods |
| Processed meat | Meatballs, fried sausage, boiled sausage, dry and cured meat, salami, jagdwurst, bologna, mortadella, ham corned beef, liverwurst, and liver pâté | G3: Processed foods |
| Cakes and sweets | Tart, pie, yeast cake, pastry, sponge cake, cream pie, cheesecake, cookies, chocolate, sweets, candy, and toffee | G4: Ultra-processed foods |
| Sweet spreads | Marmalade, jam, jelly, and honey | G4: Ultra-processed foods |
| Dairy products | Cocoa milk drink, fruit milk drink, plain yoghurt, buttermilk, flavoured yoghurt, soft cheese, semi-soft/firm cheese, sour milk, quark, mozzarella, mascarpone, feta cheese, butter, whipped cream | G4: Ultra-processed foods |
| Alcoholic beverages | Regular beer, wine, liquors, and spirits | G4: Ultra-processed foods |
| Sodas and juices | Non-alcoholic beer, sodas and minerals, light and soft drinks, fruit juices, fruit nectars, vegetable juices | G4: Ultra-processed foods |
| Vegetable soups, stews, sauces | Palmnut soup, nkontomire stew, okro stew, tomato sauce and stew, vegetable soup | Mixed classification |
| Meaty mixed dishes | Lasagne, pizza and mixed dishes with meat (fufuo ne nkatenkwan) | Mixed classification |

Table S2: Changes in percentage of total daily energy intake of Unprocessed/Minimally processed foods according to study site and socio-demographic and factors

| Socio-demographic | RURAL GHANA | | URBAN GHANA | | | AMSTERDAM | | |
| --- | --- | --- | --- | --- | --- | --- | --- | --- |
|  | % Change (95%CI) | p-value | | % Change (95%CI) | p-value | | % Change (95%CI) | p-value |
| Overall | -11.9 (-14.7- - 9.1) | **< 0.001** | | -12.0 (-14.2 - -9.7) | **<0.001** | | -0.2 (-2.0 – 1.6) | 0.828 |
| Male | -11.7(-16.0 – 7.5) | **<0.001** | | -6.1 (-9.8 - -2.4) | **0.001** | | -0.3 (-3.3 – 2.7) | 0.845 |
| Female | -12.0 (-15.6 - -8.4) | **<0.001** | | -14.4 (-17.2 - -11.6) | **<0.001** | | -0.1 (-2.3 – 2.1) | 0.906 |
| Married | -12.0 (-15.7 - -8.2) | **<0.001** | | -10.4 (-12.9 - -7.9) | **<0.001** | | 0.3 (-3.7 – 4.3) | 0.893 |
| Cohabiting | -8.4 (-14.0 - -2.8) | **0.004** | | -18.2 (-27.6 - - 8.7) | **<0.001** | | 1.0 (-2.4 -4.5) | 0.551 |
| Unmarried | -0.3 (-15.1 – 14.5) | 0.966 | | -17.8 (-26.7 - -9.0) | **<0.001** | | -0.6 (-4.7 – 3.6) | 0.782 |
| Divorced | -8.2 (-16.3 - -0.1) | **0.048** | | -11.5 (-17.6 - -5.3) | **<0.001** | | -0.7 (-4.3 – 2.8) | 0.681 |
| Widow/widower | -14.9 (-27.4 - -2.4) | **0.020** | | -20.7 (-34.5 - - 6.8) | **0.004** | | -3.6 (-29.8 – 22.6) | 0.693 |
| Never /elementary | -11.4 (-15.3 - -7.5) | **<0.001** | | -18.6 (-23.0 - - 14.3) | **<0.001** | | 0.4 (-2.8 – 3.7) | 0.787 |
| Lower voc/secondary | -9.4 (-14.3 - -4.4) | **<0.001** | | -8.1 (-10.9 - - 5.2) | **<0.001** | | -1.5 (-4.6 – 1.6) | 0.346 |
| Intermediate /second | -15.1 (-23.9 - - 6.3) | **0.001** | | -11.4 (-16.6 - -6.3) | **<0.001** | | 3.2 (0.2 – 6.1) | **0.039** |
| university | -8.8 (-26.0 – 8.4) | 0.289 | | -7.0 (-19.8 – 5.8) | 0.273 | | -4.7 (-12.4 – 2.9) | 0.212 |
| Employed | -11.0 (-14.1 - -7.8) | **<0.001** | | -12.9 (-15.6 - -10.1) | **<0.001** | | -0.4 (-3.0 – 2.2) | 0.768 |
| Unemployed | -10.3 (-18.5 - -2.2) | 0.014 | | -12.8 (-17.4 - - 8.2) | <0.001 | | 1.1 (-3.5 – 5.8) | 0.626 |

*% Change. (95%CI): Mean Percentage change (95% Confidence Interval) calculated as mean caloric contribution at follow-up minus mean caloric contribution at baseline. P-value: Paired sample t test p-value with significant values highlighted*

Table S3: Changes in percentage of total daily energy intake of processed foods according to study site and socio-demographic factors

| Socio-demographic | RURAL GHANA | | URBAN GHANA | | | | | AMSTERDAM | |  |
| --- | --- | --- | --- | --- | --- | --- | --- | --- | --- | --- |
|  | % Change (95%CI) | p-value | | % Change (95%CI) | | p-value | % Change (95%CI) | | p-value | |
| Overall | 18.8 (17.4 – 20.1) | **<0.001** | | | 9.9 (8.7 – 11.2) | **<0.001** | 5.3 (4.3 – 6.3) | | **<0.001** | |
| Male | 19.4 (17.1 – 21.6) | **<0.001** | | 10.9 (8.6 – 13.3) | | **<0.001** | 5.7 (4.0 – 7.4) | | **<0.001** | |
| Female | 18.4 (16.7 – 20.1) | **<0.001** | | 9.5 (8.0 – 11.0) | | **<0.001** | 5.0 (3.8 – 6.2) | | **<0.001** | |
| Married | 18.3 (16.4 – 20.1) | **<0.001** | | 10.5 (8.8 – 12.1) | | **<0.001** | 5.2 (3.1 – 7.3) | | **<0.001** | |
| Cohabiting | 20.7 (17.2 – 24.2) | **<0.001** | | 9.1 (3.4 – 14.8) | | **0.003** | 6.8 (4.6 – 9.0) | | **<0.001** | |
| Unmarried | 18.4 (9.7 – 27.1) | **<0.001** | | 8.0 (4.7 – 11.3 ) | | **<0.001** | 4.6 (3.0 – 6.2) | | **<0.001** | |
| Divorced | 19.3 (15.3 – 23.3) | **<0.001** | | 9.3 (5.4 – 13.3) | | **<0.001** | 5.1 (2.8 – 7.4) | | **<0.001** | |
| Widow/widower | 20.3 (16.0 – 24.7) | **<0.001** | | 9.5 (5.0 – 14.0) | | **<0.001** | 5.2 (-7.6 – 17.9) | | 0.287 | |
| Never /elementary | 18.8 (17.0 – 20.6) | **<0.001** | | 9.8 (7.7 – 11.9) | | **<0.001** | 5.9 (4.0 – 7.7) | | **<0.001** | |
| Lower voc/secondary | 19.2 (16.6 – 21.8) | **<0.001** | | 10.5 (8.6 – 12.5) | | **<0.001** | 5.2 (3.6 – 6.8) | | **<0.001** | |
| Intermediate /second | 21.1 (16.2 – 26.1) | **<0.001** | | 8.9 (5.3 – 12.5) | | **<0.001** | 6.2 (4.2 – 8.2) | | **<0.001** | |
| university | 16.8 (6.1 – 27.6) | **0.005** | | 8.0 (4.0 – 12.0) | | **<0.001** | 1.4 (-1.8 – 4.6) | | 0.372 | |
| Employed | 19.4 (17.9 – 20.8) | **<0.001** | | 10.2 (8.5 – 11.8) | | **<0.001** | 6.0 (4.6 – 7.3) | | **<0.001** | |
| Unemployed | 15.3 (9.8 – 20.8) | <0.001 | | 9.8 (7.5 – 12.1) | | <0.001 | 4.1 (1.7 – 6.5) | | 0.002 | |

*% Change. (95%CI): Mean Percentage change (95% Confidence Interval) calculated as mean caloric contribution at follow-up minus mean caloric contribution at baseline. P-value: Paired sample t test p-value with significant values highlighted*

Table S4: Changes in percentage of total daily energy intake of Ultra-processed foods according to study site and socio-demographic factors

| Socio-demographic | RURAL GHANA | | URBAN GHANA | | | AMSTERDAM | | | |
| --- | --- | --- | --- | --- | --- | --- | --- | --- | --- |
|  | % Change (95%CI) | p-value | | % Change (95%CI) | p-value | % Change (95%CI) | | p-value |  |
| Overall | -0.6 (-1.4 – 0.2) | 0.136 | | -2.0 (-3.0 - -1.1) | **<0.001** | -0.4 (-1.9 – 1.0) | | 0.539 |  |
| Male | -0.3 (-1.7 – 1.2) | 0.727 | | -3.4 (-5.4 - -1.5) | **<0.001** | -1.1 (-3.4 – 1.2) | | 0.333 |  |
| Female | -0.8 (-1.7 – 0.1) | 0.098 | | -1.5 (-2.6 - - 0.3) | **0.013** | -0.1 (-1.9 – 1.8) | | 0.980 |  |
| Married | 0.4 (-0.6 – 1.5) | 0.411 | | -2.9 (-4.2 - - 1.7) | **<0.001** | -1.0 (-4.4 – 2.3) | | 0.536 |  |
| Cohabiting | -0.8 (-2.8 – 1.1) | 0.408 | | 1.9 (-1.2 – 5.0) | 0.215 | -1.0 (-4.0 – 2.0) | | 0.495 |  |
| Unmarried | -7.1 (-13.3 - -0.9) | **0.027** | | -1.2 (-4.8 – 2.4) | 0.518 | 2.5 (-0.9 – 5.9) | | 0.144 |  |
| Divorced | -2.2 (-4.5 - - 0.2) | 0.067 | | -0.4 (-2.9 – 2.1) | 0.734 | -0.5 (-2.9 – 1.8) | | 0.641 |  |
| Widow/widower | -1.5 (-4.0 – 1.0) | 0.241 | | -2.1 (-5.3 – 1.1) | 0.198 | -17.3 (-37.1 – 2.5) | | 0.069 |  |
| Never /elementary | -0.2 (-1.2 – 0.8) | 0.753 | | -0.2 (-1.8 – 1.3) | 0.755 | 0.1 (-2.6 – 2.7) | 0.947 | |  |
| Lower voc/secondary | -1.2 (-2.7 - - 0.3) | 0.105 | | -3.7 (-5.1 - - 2.2) | **<0.001** | -1.0 (-3.6 – 1.5) | | 0.430 |  |
| Intermediate /second | -0.9 (-4.5 – 2.8) | 0.627 | | -2.0 (-5.1 – 1.2) | 0.217 | -0.4 (-3.0 – 2.2) | | 0.755 |  |
| university | 1.6 (-7.0 – 10.1) | 0.694 | | -1.3 (-5.6 – 3.0) | 0.553 | 0.1 (-5.5 – 5.6) | | 0.977 |  |
| Employed | -0.5 (-1.3 – 0.4) | 0.258 | | -2.1 (-3.3 – 0.9) | **0.001** | -1.6 (-3.7 – 0.5) | | 0.139 |  |
| Unemployed | -1.0 (-4.3 – 2.3) | 0.535 | | -2.3 (-4.1 - - 0.5) | 0.012 | 0.5 (-3.1 – 4.1) | | 0.786 |  |

*% Change. (95%CI): Mean Percentage change (95% Confidence Interval) calculated as mean caloric contribution at follow-up minus mean caloric contribution at baseline. P-value: Paired sample t test p-value with significant values highlighted*
